# The Mode of Entry of the Tubercle Bacillus

**Published:** 1908-08-15

**Authors:** 


### The Hospital

A Journal of

The Medical Sciences and Hospital Administration.

New Series. No. 75, Vol. III. [No. 1147, Vol. XLIV.] SATURDAY, AUGUST 15, 1908.

### THE MODE OF ENTRY OF THE TUBERCLE BACILLUS.

It is twenty-six years since Koch announced the discovery of the tubercle bacillus, and to-day the profession is still agitated by varying creeds concerning the avenues most commonly adopted by the organism in effecting an entry into the body. Indeed there is probably more doubt about the matter now than in the early days of the discovery, for at that time little had transpired to vex the simple belief that since the lungs were the common sites of the disease, and the sputa of tuberculous patients swarmed with the infecting agent, therefore the malady was propagated by inhalation. But presently this easy faith was troubled by the observation that the earliest lesions of tuberculosis in the lung were found in the deeper parts of the viscus: that it appeared to commence at a point accessible to inspired air only by the aid of diffusion, and inaccessible to suspended particles within that air, such as the bacilli whose inhalation and deposit seemed so straightforward an explanation of the local incidence of the disease. It became obvious, therefore, that the inspiratory theory needed qualification. Moreover with the multiplication of observations there came the demonstration of a tuberculosis which was to all appearance primary in the abdominal cavity, of another which was limited to the lymphatic glands of the neck, and of yet others in which the primary focus was remote from all possible avenues of entrance—for example, the articulation of the knee. So opinion began to rearrange itself. Koch, without definitely committing himself, inclined towards the unity of human and bovine tuberculosis, and upon the basis of this unity the profession set itself to preach the need for defence against the propagation of tuberculosis by the ingestion of tuberculous milk, thus accepting in practice, if not so readily in theory, the possibility of an intestinal path for the infection. In this tentative condition matters persisted for some years, when, in 1901, Koch startled the medical world by a positive announcement at the London Congress on Tuberculosis, that human and bovine tuberculosis were essentially distinct infections, and that for practical purposes the bovine disease was incommunicable to man. Contrary as it was to a mass of accumulated observations upon the morbid anatomy of tuberculosis in man, this announcement, coming from such a quarter, gave rise to an immense development of research. Throughout Europe private and public investigators took up the problem. The result has been the complete re-establishment of the old creed, that bovine tuberculosis is communicable to man, and that the precautions which Koch deprecated as illusory are both necessary and urgent.

It was now clear that the tubercle bacillus commands at least two possible ports of entry, the inspiratory and the alimentary canals. But the old difficulty remained unsolved. Why is it that the lungs suffer so prominently, and why, again do they suffer near the apex of the upper lobes with such peculiar frequency? It was argued that the lymphatic glands of the neck are commonly involved, and that the lymphatic system at the root of the neck may supply the path of the bacillus to the apical parts of the lung. Attention was therefore directed to the mouth and pharynx as a possible door to infection, and it was abundantly proved by numerous observations that the tonsils are capable of giving passage to tubercle bacilli, sometimes with, and sometimes without, a local lesion. Yet another observations which opens up enormous possibilities is that which demonstrated the passage of tubercle bacilli through an intact mucous membrane: for it was shown that tubercle bacilli administered to dogs could be detected in the chyle within three or four hours, a circumstance which brought under suspicion every mucous surface in the body.

With this possibility in view, attention was turned to the filtering capacity of the pulmonary parenchyma as offering an explanation of the frequency of pulmonary involvement, and recent experiments designed to demonstrate the mechanism of anthracosis (in many respects an analogue of pulmonary tuberculosis), have resulted in a good deal of illumination. A detailed account of these later researches was given by Sir William Whitla in his Cavendish lecture for the present year. The filtering power of the lung was tested by the intravenous injection of a rabbit's ear with a watery suspension of China ink. The animal was killed within an hour of the injection, and showed a well-marked pigmentation of the lungs. An oily emulsion of China ink was introduced, by means of a stomach-tube, into the stomach of an adult guinea-pig, with the result that within twelve hours of a single injection the lungs were found to be obviously pigmented, while the mesenteric glands appeared entirely free from carbon particles. A similar result attended the injection of a carbon emulsion into the peritoneal cavity, the mesenteric glands again escaping. The explanation offered is that the carbon particles effect an easy entrance into the lymphatic system, whether by means of phagocytosis or not; passing through the lacteals and lymphatic glands of the mesentery they enter the thoracic duct, are poured into the right heart, and are deposited in the fine filter supplied by the pulmonary parenchyma. When, however, young guinea-pigs were substituted for adult animals the results obtained were strikingly different. Whereas in the latter the mesenteric glands escaped after injection of a carbon emulsion into the peritoneal cavity, in the former they were blackened to a varying degree, the lungs escaping, although the costal pleura suffered. When the carbon was administered to young animals by the stomach-tube there was no notable absorption of carbon at all. Other experiments carried out with adult animals showed that ligature of the œsophagus is sufficient to prevent anthracosis after the inhalation of an atmosphere densely impregnated with carbon by means of a turpentine lamp. Also that the plugging of a bronchus in an animal exposed to a similar atmosphere does not prevent anthracosis of the lung concerned provided that the œsophagus remains open. The conclusion drawn from these and many such experiments is that anthracosis results from the ingestion of carbon, its passage through the intestinal epithelium into the thoracic duct, and its subsequent deposit in the parenchyma of the lung; and that the route of the tubercle bacillus to the lung is identical with that of the carbon granules. This is not, of course, to say that pulmonary tuberculosis is generally bovine in origin, but that the bacilli of human tuberculosis which produce the pulmonary lesions are in the first instance inhaled, are arrested in the mucous membrane of the upper passages, are swallowed, and finally reach the lung by way of the intestine. Whatever be the final judgment upon this thesis the experiments upon which it is based reflect great credit upon Vansteenberghe, Calmette, Guérin, Sir William Whitla, and the others who have elaborated them, for our old conceptions have failed to justify themselves.

